# Breast Tumor Detection and Classification Using Intravoxel Incoherent Motion Hyperspectral Imaging Techniques

**DOI:** 10.1155/2019/3843295

**Published:** 2019-07-28

**Authors:** Si-Wa Chan, Yung-Chieh Chang, Po-Wen Huang, Yen-Chieh Ouyang, Yu-Tzu Chang, Ruey-Feng Chang, Jyh-Wen Chai, Clayton Chi-Chang Chen, Hsian-Min Chen, Chein-I. Chang, Chin-Yao Lin

**Affiliations:** ^1^Graduate Institute of Biomedical Electronics and Bioinformatics, National Taiwan University, Taipei, Taiwan; ^2^Department of Medical Imaging, Taichung Tzu Chi Hospital, Buddhist Tzu Chi Medical Foundation, Taichung, Taiwan; ^3^Department of Radiology, School of Medicine, Tzu Chi University, Hualien, Taiwan; ^4^Department of Electrical Engineering, National Chung Hsing University, Taichung, Taiwan; ^5^Graduate Institute of Networking and Multimedia, National Taiwan University, Taipei, Taiwan; ^6^Department of Radiology, Taichung Veterans General Hospital, Taichung, Taiwan; ^7^Center for Quantitative Imaging in Medicine, Department of Medical Research, Taichung Veterans General Hospital, Taichung, Taiwan; ^8^Remote Sensing Signal and Image Processing Laboratory, Department of Computer Science and Electrical Engineering, University of Maryland, Baltimore County, Baltimore, MD, USA; ^9^Department of Electrical Engineering, National Taipei University of Technology, Taipei, Taiwan; ^10^Department of Breast Medical Centre, Taichung Tzu Chi Hospital, Buddhist Tzu Chi Medical Foundation, Taichung, Taiwan; ^11^Department of General Surgery, Taichung Tzu Chi Hospital, Buddhist Tzu Chi Medical Foundation, Taichung, Taiwan

## Abstract

Breast cancer is a main cause of disease and death for women globally. Because of the limitations of traditional mammography and ultrasonography, magnetic resonance imaging (MRI) has gradually become an important radiological method for breast cancer assessment over the past decades. MRI is free of the problems related to radiation exposure and provides excellent image resolution and contrast. However, a disadvantage is the injection of contrast agent, which is toxic for some patients (such as patients with chronic renal disease or pregnant and lactating women). Recent findings of gadolinium deposits in the brain are also a concern. To address these issues, this paper develops an intravoxel incoherent motion- (IVIM-) MRI-based histogram analysis approach, which takes advantage of several hyperspectral techniques, such as the band expansion process (BEP), to expand a multispectral image to hyperspectral images and create an automatic target generation process (ATGP). After automatically finding suspected targets, further detection was attained by using kernel constrained energy minimization (KCEM). A decision tree and histogram analysis were applied to classify breast tissue via quantitative analysis for detected lesions, which were used to distinguish between three categories of breast tissue: malignant tumors (i.e., central and peripheral zone), cysts, and normal breast tissues. The experimental results demonstrated that the proposed IVIM-MRI-based histogram analysis approach can effectively differentiate between these three breast tissue types.

## 1. Introduction

**Figure 1:**
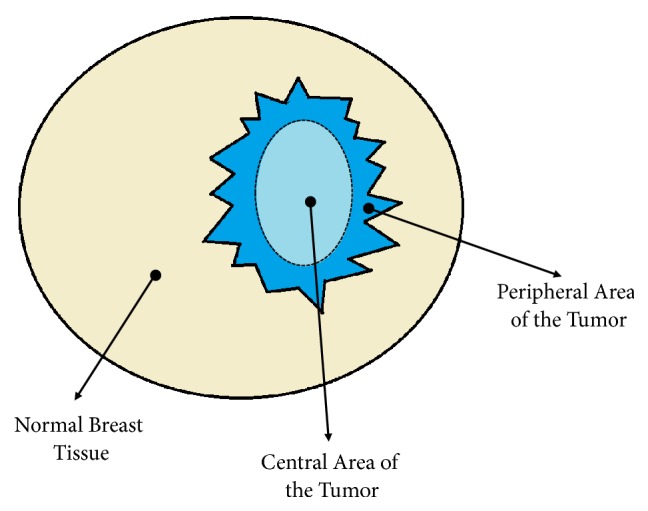
Illustration of the breast tissue regions.

Breast cancer is the most prevalent cancer and a major cause of death in women worldwide [1, 2]. Early diagnosis is critical for increasing the survival rate among women with breast cancer. However, current basic imaging approaches (e.g., mammography and ultrasonography [US]) have their limitations, especially in patients with implants, postoperative scarring, and dense breast parenchyma. These factors occasionally result in improper management of breast cancer [3–7]. Recent studies demonstrate that dynamic contrast-enhanced magnetic resonance imaging (DCE-MRI) techniques are the most promising methods for detecting tumors and assessing therapeutic response [7–9]. Furthermore, the additional diagnostic value of breast MRI is that it detects more multifocal, multicentric, and contralateral diseases [10, 11]. The sensitivity of DCE-MRI in the diagnosis of breast cancer is as high as 88%-100% [12, 13]. However, the specificity of DCE-MRI varies from 50% to 97% [11, 14–16]. This variability may result from different lesion criteria and tumor heterogeneity used among studies [15–17]. Thus, differentiating a benign lesion from a malignant lesion using DCE-MRI remains a challenge. To increase breast MRI specificity, one of the latest advancements in MRI technology, the apparent diffusion coefficient (ADC), which is obtained from diffusion-weighted imaging (DWI), was developed to differentiate between benign and malignant breast lesions with 91.5% sensitivity and 86.5% specificity [18, 19]. A typical illustration of the regions of the breast tissues is shown in Figure 1.

DWI is a nocontrast-enhanced MRI technique. It is useful for characterizing cancerous tissue heterogeneity by directly reflecting the Brownian motion of water molecules in body tissues, particularly in breast cancer [19–21]. It can obtain the physiological characteristics of breast tissue with the assumption of the monoexponential decay to quantitate the analysis of the water molecules' ADC [22]. In malignant tumors, the ADC value is usually lower than that of the normal tissue because of the restriction of water diffusion resulting from increased cellularity of the tissue and reduced extracellular space in the tumor. Based on the findings of a meta-analysis [23], the specificity of DWI is higher than contrast-enhanced MRI, although there is a wide range in threshold values from 0.90 mm^2^/s to 1.60 x 10^−3^ mm^2^/s to account for the use of different* b* values. The specificity of DWI and DCE-MRI is 84% and 72% [23].

In vivo, microscopic motion of water molecules detected by DWI is influenced by the diffusion of water molecules in the tissue structure and by the microcirculation of blood in the capillary network [24]. Signal attenuation on monoexponential DWI therefore presents a linear relationship but does not truly reflect the microstructure changes in organization. The reason for this finding is that the ADC value is overestimated due to the fact that it contains two types of information: (1) microcirculation perfusion of blood in a capillary network and (2) water molecule diffusion of the tissue structure. Intravoxel incoherent motion (IVIM), which was first described by Li Bihan et al. in 1986 [25], separates microcirculation perfusion and water molecule diffusion by using a biexponential model [22, 25, 26]. Using this biexponential model, DWI is able to use signal intensity produced by multiple* b*-values to analyze water/tissue diffusitvity. At low* b*-values, the signal intensity reflects water diffusion in tissues as well as microcirculation in a capillary network. At high* b*-values, the signal intensity reflects tissue diffusivity. Through IVIM, biomarkers have been resolved as the perfusion fraction (PF), water molecule diffusivity (D), and pseudodiffusion (D*∗*). Recent studies demonstrate that the IVIM model is helpful for the differential diagnosis of breast lesions [27–31]. In general, a MR image can be viewed as a multispectral remote sensing image, in which each spectral band image can be considered as an image acquired by a particular pulse sequence [32]. Hyperspectral imaging has become an emerging remote sensing technology that improves traditional multispectral imaging by using hundreds of contiguous spectral bands. With such fine spectral resolution, many unknown subtle substances can be revealed for subpixel detection and mixed pixel analysis [33]. The application of hyperspectral imaging techniques in MR brain imaging has also been investigated [33–35]. Many medical imaging techniques are generally supervised and require training samples provided a priori such as maximum likelihood classification (MLC), K-nearest-neighbor (KNN) classification [36], neural networks [36], support vector machine (SVM) [36], and Fisher's linear discriminant analysis (FLDA) [37]. To avoid this issue, the training samples should be obtained directly from the data to be processed in an unsupervised method. In this instance, we take advantage of the automatic target generation process (ATGP) developed by Ren and Chang [38] in conjunction with the spectral angle mapper (SAM) method, which allows users to select training samples automatically. This procedure is then followed by a supervised hyperspectral subpixel target detection technique, called kernel-based constrained energy minimization (KCEM), developed by Jiao and Chang [39], to detect breast lesion tissues for quantitative analysis for IVIM-DWI. This technique considers MR image slices acquired by pulse sequences and DWI with weighting factors of 12* b*-values as a 12-band multispectral image cube in which each sequence-acquired image is viewed as a spectral band image. The data set to be used for experiments was collected from 25 patients for IVIM imaging examinations.

The purpose of this study was three folds: (1) to measure IVIM parametric maps (i.e., tissue diffusivity [D], perfusion fraction [PF], pseudodiffusion coefficient [D*∗*], and slope) obtained from multiple* b* values of normal breast tissue, cysts, and malignant tumors; (2) to determine whether these parameters can be used to distinguish between benign and malignant breast lesions; and (3) to calculate advanced diffusion MRI metrics from histogram analysis (i.e., mean and median), and heterogeneity.

## 2. Background

### 2.1. Diffusion-Weighted Imaging

Diffusion-weighted imaging (DWI) is sensitive to the thermally driven random motion of water molecules. Molecular diffusion is the random motion of molecules, called Brownian motion [25, 40]. The motion is modified by the local tissue environment and the cell membranes. The motion of water molecules is more restricted in tissues with a high cellular density than in tissues with low cellularity. Signal intensity in DWI is inversely proportional to the degree of water molecule diffusion. The histologic structure is influenced by diffusion. The change of water diffusion in tissues is useful for MR imaging and can be used as a multifaceted tool to characterize tissue structures and identify and differentiate disease processes. Furthermore, DWI can be used to calculate the ADC, which can quantitatively measure the degree of tissue diffusivity.

Spin-echo echo-planar DWI is the most popular clinical technique to produce diffusion-weighted images in which diffusion is described by the following monoexponential equation [25]:

(1) $$\begin{array}{c} \ln\left(\;\frac{S_{b}}{S_{0}}\;\right)=-b\left(\;ADC\;\right)\Leftrightarrow ADC=-\ln\frac{\left(S_{b}/S_{0}\right)}{b} \end{array}$$

with

(2) $$\begin{array}{c} b=\gamma^{2}g^{2}\delta^{2}\left(\;\Delta -\frac{\delta }{3}\;\right) \end{array}$$

in which* S*_b_ is DWI with diffusion attenuation at the* b* value and *S*_0_ is DWI without diffusion attenuation, *g* is the diffusion gradient, *γ* is the proton gyromagnetic ratio, and *δ* is the diffusion gradient duration. Equation (2) presents* b* value (expressed in seconds per square millimeter) and represents the strength of diffusion weighting in which* S*_b_ is the signal intensity with the diffusion gradient.* S*_0_ is the signal intensity without the diffusion gradient. The monoexponential model is traditionally used to calculate the decay of ADC in (2) and results from two* b* values,* b*_1_ and* b*_2_, as follows:

(3) $$\begin{array}{c} D=-\frac{1}{b_{1}-b_{2}}\ln\left(\;\frac{S\left(b_{1}\right)}{S\left(b_{2}\right)}\;\right) \end{array}$$

### 2.2. Intravoxel Incoherent Motion Imaging

Using the value of ADC in (3) does not sufficiently describe tissue behavior. To address this issue, Le Bihan et al. [25] proposed a novel approach, called IVIM MRI, which demonstrated that the ADC values of data samples obtained at low* b* values can be used to measure two IVIM parameters: (1) pseudodiffusion (D*∗*) and (2) the perfusion fraction (PF) of pure molecular diffusion and microcirculation, or blood perfusion [25, 26]. In particular, D*∗* and PF can be used as quantitative biomarkers to describe changes in diffusivity and microcapillary perfusion of tissues. Increasing the* b*-value can change the values of D*∗* and PF and therefore can reflect the diffusivity of different tissues and tissue microcapillary perfusion.

**Figure 2:**
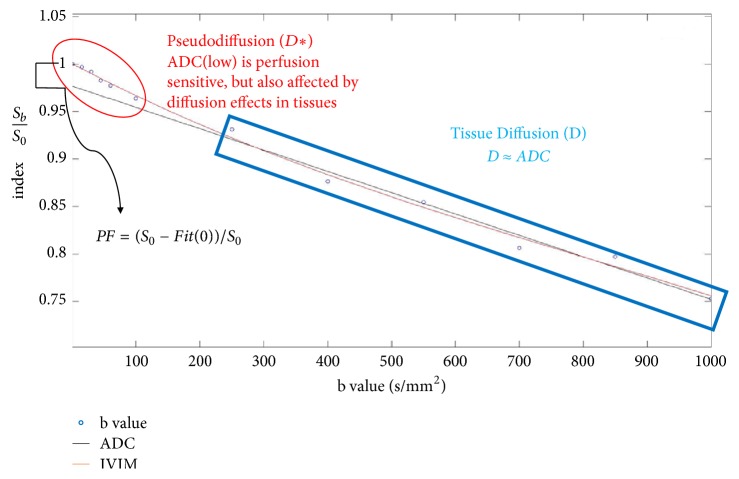
The graph of curve fitting of IVIM imaging used the biexponential model to separate the estimation of tissue diffusivity and perfusion based on multiple *b* values.

DWI is influenced by the diffusion of water molecules, which is affected by microcapillary blood in the capillary network in tissue structures. Both processes cause phase dispersion in DWI, and consequently signal attenuation. Le Bihan et al. [25] described the behavior of protons that displayed signal attenuation in DWI as IVIM imaging, which does not represent a linear relationship as shown in Figure 2. The biexponential model involves curve fitting for IVIM imaging to separate the estimation of tissue diffusivity from perfusion by using multiple* b* values, in the following equation:

(4) $$\begin{array}{c} \frac{S_{b}}{S_{0}}=\left(\;1-PF\;\right)\exp\left(\;-bD\;\right)+PF\exp\left(\;-bD*\;\right) \end{array}$$

where D is the pure diffusion coefficient, S_b_ is the signal intensity in the pixel with diffusion gradient, and S_0_ is the signal intensity in the pixel without a diffusion gradient. This biexponential analysis, based on the IVIM sequence, can be used to describe the mixture of perfusion and diffusion resulting from DWI in three parameters: pure diffusion coefficient [D], perfusion fraction [PF], and pseudodiffusion coefficient [D*∗*].

### 2.3. Band Expansion Process

Intravoxel incoherent images do not provide sufficient spectral information to be processed as hyperspectral images; therefore, the band expansion process (BEP) developed by Ren and Chang [41] is used to resolve this issue for IVIM analysis. This process produces new images from the original IVIM images via nonlinear functions. Its premise is similar to the moment generating function used by a random process that makes use of the statistics of all orders to describe probabilistic behaviors of a random process. In the BEP, only the statistics of the second order of IVIM images, autocorrelation, and cross-correlation functions are used for this purpose. Such nonlinearly generated images provide nonlinear spectral information contained in IVIM images and can help improve data analysis.

The BEP presented in this section is a nonlinear process using correlation functions to generate new band images from the original set of multispectral images.

The BEP for IVIM images is as follows.

**Step 1 .**

The 1^st^-order IVIM images are obtained as follows: _{*B*_*l*_}_*l*=1_^*L*^ = the set of original IVIM images

**Step 2 .**

The 2^nd^-order correlated IVIM images are obtained as follows:

- {**B**_*l*_^2^}_*l*=1_^*L*^= the set of autocorrelated IVIM images;
- {**B**_*k*_**B**_*l*_}_*k*=1,*l*=1,*k*≠*l*_^*L*,*L*^ = the set of cross-correlated IVIM images.

In case a rescaling is needed, auto- or cross-correlated IVIM images can be normalized by variances in the IVIM images such as (*σ*_*B*_*l*__^2^)^−1^{*B*_*l*_^2^} and (*σ*_*B*_*k*__*σ*_*B*_*l*__)^−1^{*B*_*k*_*B*_*l*_}.

**Step 3 .**

The 3^rd^ order correlated IVIM images are obtained as follows:

- {**B**_*l*_^3^}_*l*=1_^*L*^ = the set of autocorrelated IVIM images;
- {**B**_*k*_^2^**B**_*l*_}_*k*=1,*l*=1,*l*≠*k*_^*L*,*L*^ = the set of two cross-correlated IVIM images;
- {**B**_*k*_**B**_*l*_**B**_*m*_}_*k*=1,*l*=1,*m*=1,*k*≠*l*≠*m*_^*L*,*L*,*L*^= the set of three cross-correlated IVIM images.

As in Step 2, auto- or cross-correlated IVIM images can be similar. Therefore, it may require normalization by the variances in the IVIM images. For example,

(5) $$\begin{array}{c} {\left(\;\sigma_{B_{l}}^{3}\;\right)}^{-1}\left\{\;B_{l}^{3}\;\right\},\\ {\left(\;\sigma_{B_{k}}^{2}\sigma_{B_{l}}^{\;}\;\right)}^{-1}\left\{\;B_{k}^{2}B_{l}\;\right\},\\ {\left(\;\sigma_{B_{k}}^{\;}\sigma_{B_{l}}^{\;}\sigma_{B_{m}}^{\;}\;\right)}^{-1}\left\{\;B_{k}B_{l}B_{m}\;\right\}. \end{array}$$

**Step 4 .**

Nonlinear correlated IVIM images are obtained as follows:

- $\{\sqrt{B_{l}}{\}}_{l=1}^{L}$ = the set of IVIM images stretched out by the square root;
- $\{\log\sqrt{B_{l}}{\}}_{l=1}^{L}$ = the set of IVIM images stretched out by the logarithmic function.

**Figure 3:**
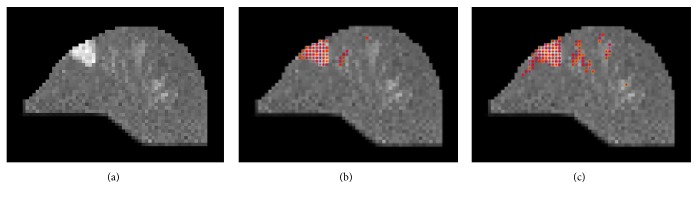
An example of using BEP to generate left breast malignant tissue in (a) ground truth (b) an image compared to image (c), and (c) not using BEP.

Figure 3 shows an example of IVIM images (a) ground truth (b) compared to the one in (c) without using BEP.

### 2.4. Automatic Target Generation Process

Once IVIM images are nonlinearly expanded, they are then processed by an unsupervised target detection algorithm, called automatic target detection algorithm (ATGP), to detect potential unknown targets without prior knowledge. The procedure is briefly described as follows.

Let** X** be a data matrix formed by data sample vectors,{**r**_*i*_}_*i*=1_^*N*^, (i.e., **X** = [**r**_1_**r**_2_ ⋯ **r**_*N*_].)The norm of the data matrix** X** is defined by the equation, as follows:

(6) $$\begin{array}{c} \left\|\;X\;\right\|=\underset{1\leq i\leq N}{\max}\left\|\;r_{i}\;\right\| \end{array}$$

where ‖**r**_*i*_‖ is the length of the vector **r**_*i*_ = (*r*_*i*1_, *r*_*i*2_, ⋯,*r*_*iL*_)^*T*^ as defined by ‖**r**_*i*_‖^2^ = ∑_*l*=1_^*L*^*r*_*il*_^2^ and assuming that *i*^*∗*^ = arg⁡ {max_1≤*i*≤*N*_⁡‖**r**_*i*_‖}. The norm of data matrix** X** in (6) can further be expressed by

(7) $$\begin{array}{c} \left\|\;X\;\right\|=\left\|\;r_{i^{*}}\;\right\| \end{array}$$

which is exactly the brightest pixel **r**_*i*^*∗*^_ and its norm has the maximum vector length. The maximum* l*_2_ norm defined by (7) is indeed the maximum pixel vector length corresponding to the brightest pixel vector in the data set.

Using (7), the ATGP produces a sequence of orthogonal subspace projections (OSPs) as follows:

(8) $$\begin{array}{c} P_{U}^{⊥}=I-UU^{\#} \end{array}$$

Thus, if** X** is the original hyperspectral image cube, the ATGP first selects an initial target pixel *t*_0_^*ATGP*^ that yields the norm of the space** X**, denoted by ‖**t**_0_^ATGP^‖ = ‖**X**‖ via (6). It then projects the space** X** into a subspace orthogonal to 〈**t**_0_^ATGP^〉 via *P*_**U**_0__^⊥^ with **U**_0_ = [**t**_0_^ATGP^]. The resulting subspace is denoted by **X**_1_ = 〈**t**_0_^ATGP^〉^⊥^. The ATGP selects a first target pixel that yields the norm of space** X**_1_, denoted by ‖**t**_1_^ATGP^‖ = ‖**X**_1_‖ via (7), and then projects space** X** into a subspace orthogonal to 〈**t**_0_^ATGP^, **t**_1_^ATGP^〉 via *P*_**U**_1__^⊥^ with **U**_1_ = [**t**_0_^ATGP^**t**_1_^ATGP^]. The resulting subspace is denoted by **X**_2_ = 〈**t**_0_^ATGP^, **t**_1_^ATGP^〉^⊥^. The same procedure is repeated until the stopping rule is satisfied, (i.e., the number of target pixels for the ATGP required to extract). The details of its algorithmic implementation are as follows.

(1) Initial condition: the variable *ε* is the prescribed error threshold, and **t**_0_ is a pixel with the brightest intensity value (i.e., the maximal gray level value.) Set* k=0*.

(2) For *k* ← *k* + 1, *P*_**t**_0__^⊥^ is applied via (8) with** U**=[**t**_0_] to all image pixels;** r** in the image. The *k*^th^ target** t**_k_ is generated at the *k*^th^ stage, which has the maximum orthogonal projection, as follows:

(9) $$\begin{array}{c} t_{k}=\arg\left\{\;\underset{r}{\max}\left[\;{\left(\;P_{\left[U_{k-1}t_{k}\right]}^{⊥}r\;\right)}^{T}\left(\;P_{\left[U_{k-1}t_{k}\right]}^{⊥}r\;\right)\;\right]\;\right\} \end{array}$$

If *m*(**t**_*k*−1_, **t**_*k*_) > *ε* in which *m*(·, ·) can be any target discrimination measure, then Step 2 is repeated. The algorithm is otherwise terminated. At this point, all generated target pixels **t**_0_, **t**_1_, ⋯, **t**_*k*−1_ are the desired targets.

### 2.5. Spectral Angle Mapper

The targets generated by ATGP are single targets. To find other targets that have spectral signatures similar to those of the ATGP-detected targets, we used the SAM [42] as a spectral measure to calculate the similarity between two vectors by finding their angles [33]. In particular,** s** and** t **were two vectors. The SAM is defined by

(10) $$\begin{array}{c} SAM\;\left(\;s,t\;\right)=cos^{-1}\;\left(\;\frac{s^{T}t}{{\left\|s\right\|}^{1/2}{\left\|t\right\|}^{1/2}}\;\right)\;\;in\;\;radians \end{array}$$

In general, it is used to compare a spectral vector against a reference vector in terms of their between angle. Therefore, the larger the angle is in (10), the less is the similarity.

### 2.6. Kernel-Based Constrained Energy Minimization

Once targets were determined and identified by using SAM, a target detector was further designed for target detection. In this section, a finite impulse response (FIR) linear filter, called constrained energy minimization (CEM) developed in several studies [42–44], was used for this purpose.

More specifically, CEM is derived from the linearly constrained minimum variance originally proposed by Frost for adaptive beam forming [45]. If a hyperspectral image is represented by a collection of image pixel vectors, denoted by {**r**_1_, **r**_2_, ⋯, **r**_*N*_} in which **r**_*i*_ = (*r*_*i*1_, *r*_*i*2_, ⋯,*r*_*iL*_)^*T*^ for 1 ≤ *i* ≤ *N* is an* L*-dimensional pixel vector, then* N* is the total number of pixels in the image and* L* is the total number of spectral channels. Furthermore, **d** = (*d*_1_, *d*_2_, …,*d*_*L*_)^*T*^is specified by a desired signature of interest to be used for target detection. The goal is to find a target detector that can detect data samples specified by the desired target signal** d** via a FIR filter with* L* filter coefficients, {*w*_1_, *w*_2_, ⋯, *w*_*L*_}, denoted by an* L*-dimensional vector **w** = (*w*_1_, *w*_2_, ⋯,*w*_*L*_)^*T*^, which minimizes the filter output energy, subject to the constraint **d**^*T*^**w** = **w**^*T*^**d** = 1. More specifically, if* y*_i_ denotes the output of the designed FIR filter resulting from the input r_i_, then* y*_i_ can be expressed by the following equation:

(11) $$\begin{array}{c} y_{i}=\overset{L}{\underset{l=1}{\sum }}w_{l}r_{il}={\left(\;w\;\right)}^{T}r_{i}=r_{i}^{T}w \end{array}$$

The average energy of the filter output is given by (12), in which (13) provides the autocorrelation sample matrix of the image:

(12) 1N∑i=1Nyi21N∑i=1NriTw2=wT1N∑i=1NririTw=wTRw

(13) R1N∑i=1NririT

The goal was to solve the following linearly constrained optimization problem:

(14) minw wTRwsubject  to dTw=wTd=1

in which **w**^*T*^**RW** is the variance resulting from signals not passing through the filter. The optimal solution to (14), as described in [17–19], is presented in (15) and (16), as follows:

(15) wCEM=dTR−1d−1R−1d

(16) minw⁡ wR−1w=wCEMTR−1wCEM=1dTR−1d

which is the CEM error considered as the least energy resulting from unwanted signal sources impinging on an array of sensors. With the optimal weight, **w**^CEM^, specified by (15), a filter, called CEM and denoted by *δ*^CEM^(**r**), was derived from a previous study [43] and can be specified by

(17) $$\begin{array}{c} \delta^{CEM}\left(\;r\;\right)={\left(\;w^{CEM}\;\right)}^{T}r={\left(\;d^{T}R^{-1}d\;\right)}^{-1}{\left(\;R^{-1}d\;\right)}^{T}r \end{array}$$

Constrained energy minimization is a linear filter and IVIM images are nonlinearly generated multispectral images; therefore, nonlinear separability may present an issue for CEM. To mitigate this problem, CEM was further expanded to a kernel version of CEM, called kernel CEM (KCEM), which was derived as follows [39]: Φ : *ℜ*^*N*^ → *F*  by  **r** ↦ Φ(**r**). The** R** in (13) can be expanded in feature space* F* as presented in

(18) $$\begin{array}{c} R_{\Phi }=\frac{1}{N}\overset{N}{\underset{i=1}{\sum }}\Phi^{T}\left(\;r_{i}\;\right)\Phi \left(\;r_{i}\;\right) \end{array}$$

The CEM derived from (17) can be extended to a kernel version of CEM (KCEM), given by

(19) $$\begin{array}{c} \delta^{K\text{-}CEM}\left(\;r\;\right)=\frac{\Phi^{T}\left(d\right)R_{\Phi }^{-1}\Phi \left(r\right)}{\Phi^{T}\left(d\right)R_{\Phi }^{-1}\Phi \left(d\right)} \end{array}$$

By virtue of (19), the KCEM can be calculated in feature space* F* via the kernel trick without mapping the original data sample vectors into the feature space. Several commonly used kernel functions can be used for (19). In this paper, the Gaussian-based radial basis function (RBF) kernel is given by

(20) $$\begin{array}{c} k\left(\;x,y\;\right)=\exp\left(\;\frac{-{\left\|x-y\right\|}^{2}}{2\sigma^{2}}\;\right),\;for\;\;\sigma \in R \end{array}$$

which was used for experiments to implement KCEM. There are two reasons for choosing Gaussian-based RBF kernel: (1) it is a translation invariant kernel, and (2) its associated nonlinear map is smooth. Even when the spectral signatures of a given hyperspectral data set are subject to irregular illumination, the translationally invariant kernels typically provide robust detection. This finding is because it depends only on the difference between** x **and** y**, not the absolute positions of a single spectral vector. The smooth nonlinear mapping associated with the Gaussian RBF kernel means that, after nonlinear mapping, the topographic ordering of the data in the input space is preserved in the feature space. The mapped data sample vectors in the feature space also occupy the small subspace of the feature space, in which data belonging to different classes can be separated to a larger extent than in the input space. As a result, the Gaussian RBF kernel-mapped feature space in the target detection task is more effective.

### 2.7. Thresholding

The KCEM-generated detection map is real valued; therefore, it requires a thresholding technique to produce a binary image to show detected lesion tissues. In the literature, many thresholding techniques have been proposed such as Otsu's method [46], two entropic thresholding methods, local entropy and joint entropy methods [45], and three relative entropy methods, local relative entropy, joint relative entropy, and global relative entropy methods in [47, 48]. Based on our extensive experiments, the local entropy method [45] was the best among entropy-based methods and outperformed the most widely used Otsu's method. The local entropy method was consequently selected as the thresholding technique to segment lesions from the KCEM-produced detection maps.

### 2.8. Decision Tree

A decision tree is a nonparametric supervised learning method used for classification and regression. It builds classification or regression models in the form of a tree structure, in which each internal node denotes a test on an attribute, each branch represents an outcome of the test, and leaf nodes represent classes or class distributions. It breaks a dataset into smaller and smaller subsets while incrementally developing an associated decision tree at the same time.

Decision trees can be used for categorical and numerical data. The goal is to create a model that can be used to predict the value of a target variable by learning simple decision rules inferred from the data features.

The lesions detected by the local entropy-thresholder KCEM were further used as the inputs of a decision tree. In order to perform breast lesion classification, the decision tree is specifically developed by a nonparametric supervised learning method, which is generally used for classification and regression. It builds classification or regression models in the form of a tree structure, in which each internal node denotes a test based on an attribute. Each branch represents an outcome of the test, and leaf nodes represent classes or class distributions. Categorical and numerical data can be used in such decision trees. The goal was to create a model that could be used to predict the value of a target variable by learning simple decision rules inferred from the data features.

## 3. Materials and Methods

### 3.1. Patient Selection

After obtaining institutional review board approval to conduct this prospective study, 25 patients with breast cancer (20 malignant lesions and 5 cysts) underwent MR examination in our institution from May 2014 to June 2015. Written informed consent was obtained from all patients. The patient inclusion criteria were as follows: (1) newly diagnosed with breast cancer confirmed by needle biopsy; (2) the patient was not receiving neoadjuvant chemotherapy or hormonal treatment at the time of imaging; (3) tumor size (solid portion, referring to the T2-weighted and contrast-enhanced T1-weighted images)> 1 cm in DW imaging; (4) unilateral breast cancer; (5) no previous breast surgery history, and (6) no motion artifact. The definitive diagnosis of cyst was obtained by means of ultrasonographic (US) and breast MR imaging findings. The simple cyst shows thin wall, circumscribed margin and anechoic at US, low T1 signal intensity, high T2 signal intensity at fat-suppressed images, and no enhancement at postcontrast fat-suppressed images. All MRI studies were reviewed by an experienced radiologist (S.W.C) who had access to all patient information.

### 3.2. Experimental Materials

MRI was conducted using a 1.5T system (Aera; Siemens, Erlangen, Germany) using a body coil as the transmitter and a dedicated 16-channel receiver coil. The patient was prone and head first. Conventional T1- and T2-weighted images were acquired with and without fat suppression. The axial IVIM images were obtained by using single-shot spin-echo echo-planar imaging (EPI). The axial IVIM images with bilateral breast coverage were acquired (TR/TE: 5800/68 ms; FOV: 320 mm^2^ x 264 mm^2^; matrix: 132 x 160 x 30; reconstructed voxel size: 2 mm^3^×2 mm^3^×3 mm^3^; with spectral presaturation inversion recovery and diffusion sensitization in the anterior-posterior direction applied with weighting factors of* b* = 0, 15, 30, 45, 60, 100, 250, 400, 550, 700, 850, 1000 sec/mm^2^. Total scan time required by an IVIM imaging scan was 6.3 minutes. Axial T1-weighted DCE_MRI (TR/TE, 4.5/1.8 ms; FA, 12; FOV, 320 mm^2^ x 320 mm^2^; matrix size, 512 x 512; slice thickness, 1.5 mm) was acquired using the gradient echo sequence. The contrast media were captured after precontrast and four consecutive time points (i.e., 60-second interval time) after administrating of gadolinium (Gadovist, 1.0 mmol/mL) by using a power injector, at a flow rate of 2.0 mL/s, followed by 20 cc normal saline flush.

Sixteen sets of MR images were used for the experiments, which were acquired by spin-lattice relaxation time (T1), spin-spin relaxation time (T2), proton density (PD), and T1-DCE along with IVIM based DW images, for which T1-DCE was involved using contrast agents at different times. These IVIM-DWI images were considered as the multispectral images.

Image analysis was conducted on a personal computer using in-house software written in MATLAB (Math Works, Natick, MA). The IVIM parameters were calculated from the DW images at all* b*-values by using the biexponential model, described by Le Bihan et al. [25, 26]. At first, the assumption parameters derived from the IVIM image using all* b*-values were compared with DCE_MRI to obtain reliable results. A radiologist with 13 years of experience read all MRI breast images to determine the tumor region of interest (ROI) and exclude areas of necrosis, bleeding, and edema. After, parameters were mapped again onto the DCE_MRI as the ground truth. There is no need of ROI in our study because entire images were processed, pixel by pixel. Therefore, interobserver variability was limited.

### 3.3. Preprocessing

Before processing the data, a technique, called nonparametric nonuniform intensity normalization (N3), was used to adjust the nonuniformity of the MR images. Multiple* b*-values images of IVIM displacement caused by respiration must be corrected; therefore, an alignment. was used to register all pixels. Once all images were aligned, an automatic method to extract the breast region was developed, as follows. Automatic process to extract the breast region:

  Step 1. A high pass filter was used to enhance the boundary of the breast image.
  Step 2. A Sobel edge detector was used to extract the contour of the breast image.
  Step 3. The hit-or-miss transformation function was applied to the flat area between two breasts to extract the sternum.
  Step 4. Otsu's method was implemented to produce a binary image.
  Step 5. A morphological opening using a structural element with a window (size, 2x2 pixels) was used to detach the connected components in the binary image.
  Step 6. A morphological opening was used to isolate the largest connected component.
  Step 7. A Sobel edge detector was applied again to obtain the silhouette of the breast image.

### 3.4. Breast Lesion Tissue Detection

The BEP was used to create additional IVIM images for the IVIM-MRI classification. After BEP, the original images were expanded to 90 band images. Since KCEM requires the knowledge of the desired signature** d**, therefore, we first used ATGP to determine the initial target pixels. These pixels were further used to find target pixels with similar spectral signatures measured by SAM as the training samples that could be used as the desired** d** for KCEM. The detected region was further verified by an experienced breast radiologist and by tumor biopsy. The detected part of each breast lesion tissue on an ADC map was calculated by all* b* values by using (3). For all lesion tissues, the D, D*∗*, PF, and slope values were further calculated on a pixel-by-pixel basis by using (4). The lesion tissues of 25 individual patients were tested and the statistical values of all parameters were used as the standard for the decision tree.

### 3.5. Quantitative Analysis

This study was conducted on IVIM-DWI using 12* b* values:* b*=0, 15, 30, 45, 60, 100, 250, 400, 550, 700, 850, 1000 s/mm^2^. The biexponential model was proposed by Le Bihan et al. [25]; microcapillary perfusion of the blood has no specific orientation, which depends on the velocity of flowing blood and the vascular architecture. The effect of pseudodiffusion on signal attenuation in an IVIM-DWI voxel is also* b* value dependent. The biexponential analysis to describe DWI comprises a mixture of perfusion and diffusion.

The analytic model of IVIM is a biexponential and monoexponential model, demonstrated in (4). The monoexponential model of IVIM is expressed in (1). The ADC was obtained by using all* b*-values (0-1000 s/mm^2^) and then fitted to the (4). Four parameters, D (pure molecular diffusion), D*∗* (perfusion-related diffusion), PF (perfusion fraction), and ADC (apparent diffusion coefficient), were determined after undergoing processing by image preprocessing, detection, and analysis of the lesion. Through these parameters, the lesion area could be further compared.

### 3.6. Histogram Analysis

In this section, a quantitative analysis was conducted on the IVIM-DWI images obtained by using 12 diffusion-weighted* b *values 0, 15, 30, 45, 60, 100, 250, 400, 550, 700, 850, 1000 s/mm^2^. The faster a molecule diffuses, the greater is the attenuation and the weaker is the corresponding pixel signal intensity on a DWI image. The biexponential analysis used in. (4) to describe DWI comprises a mixture of perfusion and diffusion, whereas a simple monoexponential analysis only uses the* b*-values of 0 s/mm^2^ and 1000 s/mm^2^ via (3).

**Figure 4:**
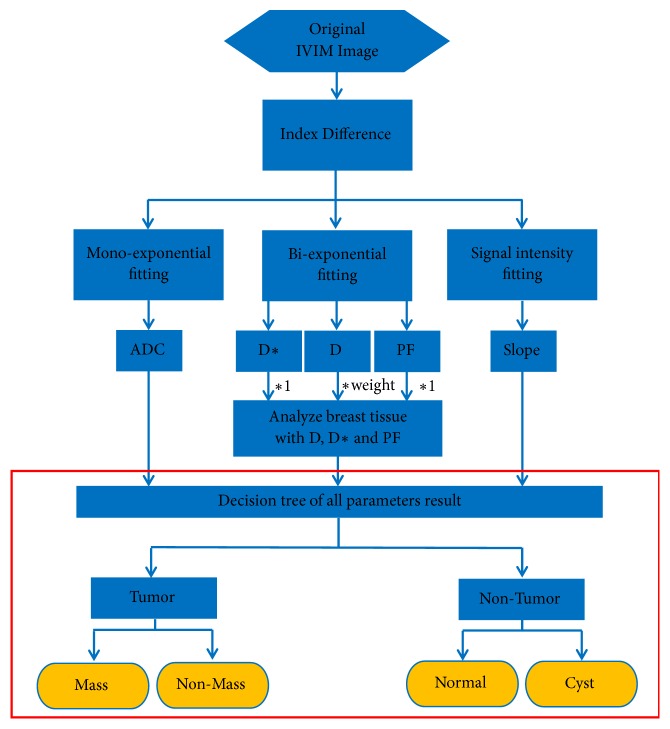
The flow chart shows the decision tree structure for breast tissue classification using ADC, D, D*∗*, PF, and slope parameters.

The ADC was obtained by the monoexponential model in (3) using* b*-values ranging from 0 to 1000 s/mm^2^ and then fitted to the biexponential model in (4). Four parameters result from the series of processes, preprocessing, detection, and analysis of the lesion tissue: D (pure molecular diffusion), D*∗* (perfusion-related diffusion), PF (perfusion fraction), and slope.  Figure 4 shows the flowchart for IVIM imaging processing.

Details of step-by-step implementations are as follows.

**Step 1 .**

The ADC, D, D*∗*, PF, and slope values were calculated to find the threshold values for central tumor, peripheral tumor, normal tissue, and cyst.

**Step 2 .**

The ADC, slope, D, D*∗*, and PF values of each pixel were used to run the decision tree to determine to which of the three categories a pixel belongs to: tumor, normal tissue, or cyst.

**Step 3 .**

If a pixel indicated a tumor, then histogram analysis was used to determine whether the pixel indicated a central tumor or a peripheral tumor.

**Step 4 .**

Otherwise, the pixel indicated a nontumor lesion. The decision tree was then run again to determine whether the pixel was normal tissue, cyst, or other.

Histogram analysis can provide information beyond the IVIM parameters in the spatial distribution histogram, such as the skewness and kurtosis of the parameter distributions. Skewness is a measure of symmetry, where kurtosis is a measure of whether the data are peaked or flat relative to a normal distribution.

## 4. Results and Discussion

**Figure 5:**
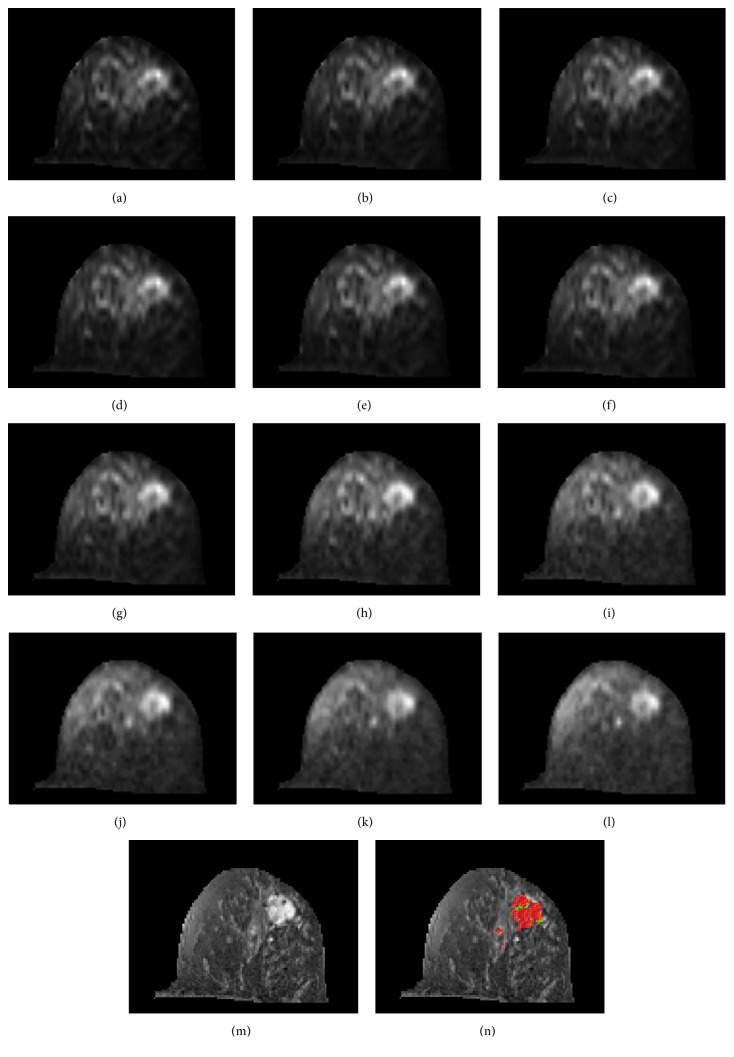
The IVIM-DWI images of left breast cancer at different* b* values: (a)* b *= 0 (b)* b *=15 (c)* b* = 30 (d)* b* = 45 (e) b = 60 (f)* b* = 100 (g)* b* = 250 (h)* b* = 400 (i)* b* = 550 (j)* b* = 700 (k)* b* = 850 (l)* b* = 1000 s/mm^2^. (m) The dynamic T1-DCE MR image. (n) Lesion mapping with red and green pixels indicating the central and peripheral tumor regions, respectively.

Figure 5 shows a case of breast cancer on IVIM images at different* b* values: (a)* b *= 0 (b)* b *=15 (c)* b* = 30 (d)* b* = 45 (e) b = 60 (f)* b* = 100 (g)* b* = 250 (h)* b* = 400 (i)* b* = 550 (j)* b* = 700 (k)* b* = 850 (l)* b* = 1000 s/mm^2^. (m) The dynamic T1-DCE MR image. The lesion mappings are shown in image (n). The red and green pixels indicate the central tumor region and the peripheral tumor region, respectively.

**Figure 6:**
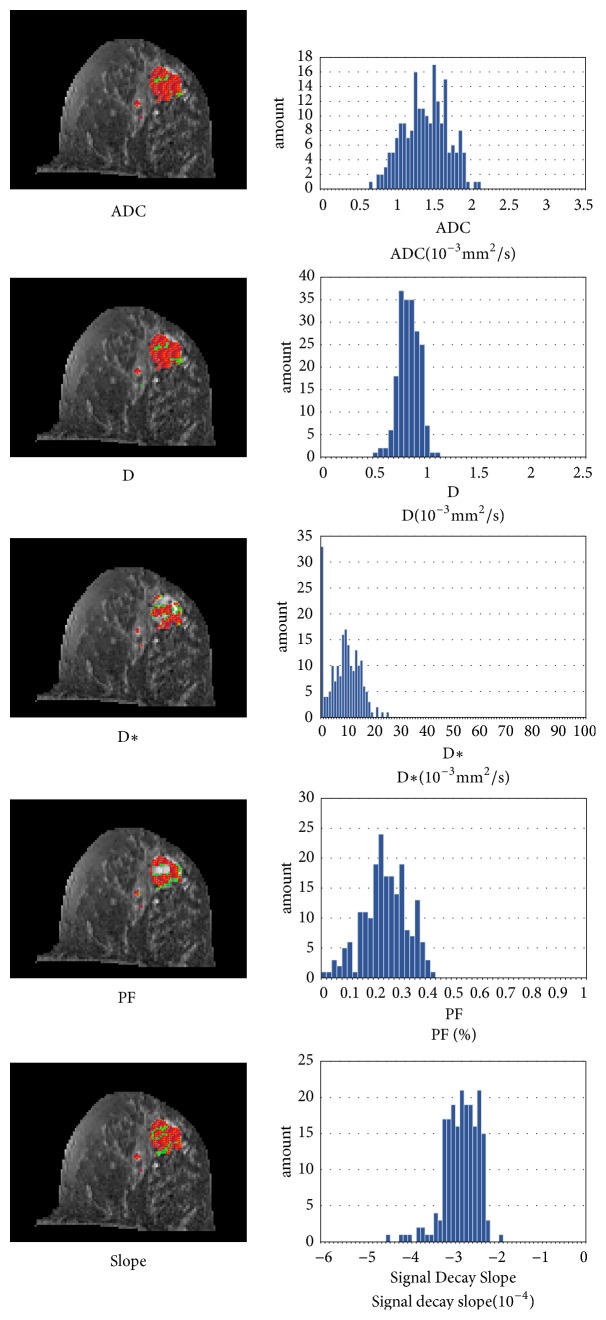
The ADC, D, D*∗*, PF, and slope parameters of the histogram analysis in the range of the tumor. The red and green pixels indicated the central and peripheral tumor regions, respectively.

Figure 6 shows the ADC, D, D*∗*, PF, and slope values of the histogram analysis in the range of the tumor. The red and green pixels show the central tumor and peripheral tumor region, respectively.

**Figure 7:**
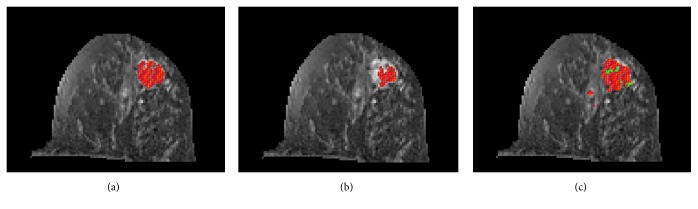
(a) Lesions detected by KCEM, (b) lesions detected by thresholding the image in (a) by using local entropy method, (c) lesion detection by histogram analysis using decision tree.

To automatically find potential lesion pixels, ATGP was used to find the brightest point, and then the SAM algorithm was applied to locate the points with spectral signatures similar to the ATGP-found point that could be used as desired target signatures for KCEM to detect a suspected tumor range. However, the signal strength was insufficiently strong to be curbed and the tumor area could not be fully detected. Therefore, a local entropic thresholding method was applied to KCEM for suspected tumor range improvement.  Figure 7(a) shows the results of tumor detection using KCEM.

The local entropy method was used to find an appropriate threshold value. Threshold results are presented in Figure 7(b) in which areas with low intensity values could still be detected by KCEM. However, using the local entropy method to adjust threshold values could only capture areas that are different from the background. In addition, in this technique, the threshold value needs to be manually adjusted slice by slice; thus, it is also subjective and time consuming. To address this issue, a histogram analysis using the five parameters of ADC, D, D*∗*, PF, and slope was further used to determine the true scale of the suspected tumor area. As a result, some dense parts of the tissues could also be detected, as shown in Figure 7(c).

The monoexponential and biexponential models [i.e., (3) and (4), respectively] were then applied to the IVIM-DW images in which the relative signal intensity decays of central tumor, peripheral tumor, cyst, and normal tissue are specified by the five parameters of ADC, D, D*∗*, PF, and slope. We first obtained MRI-IVIM images and then obtained contrast-enhanced MR images as the ground truth. We used a patient with breast cancer to demonstrate how these five parameters could be used to classify breast tissues. In Figure 7(b), the tumor area was marked by red pixels and was detected by the KCEM method using the local entropy method. For the pixels in the red area, the calculated values of D*∗* were larger than those of other areas without tumor. The D and ADC values in the tumor area were both lower than those in the normal breast tissue area. The D, PF, and D*∗* values obtained from IVIM biexponential model fitting and the ADC value obtained from monoexponential model fitting were also used to classify breast tissues into three categories, the tumor (central and peripheral tumor), cyst, and normal tissues. These values are tabulated in Tables 1 and 2.

In particular, Table 2 tabulates the average results of ADC, D, D*∗*, PF, and slope parameters of the total cases (i.e., test results of 25 patients) based on mono- and biexponential model analysis. For all images, each slice contained 12 IVIM images from b=0 mm^2^/s to b=1000 mm^2^/s. Each case may contain 20 IVIM slices and more than 40 T1, T2, PD, and T1-delay slices.

**Figure 8:**
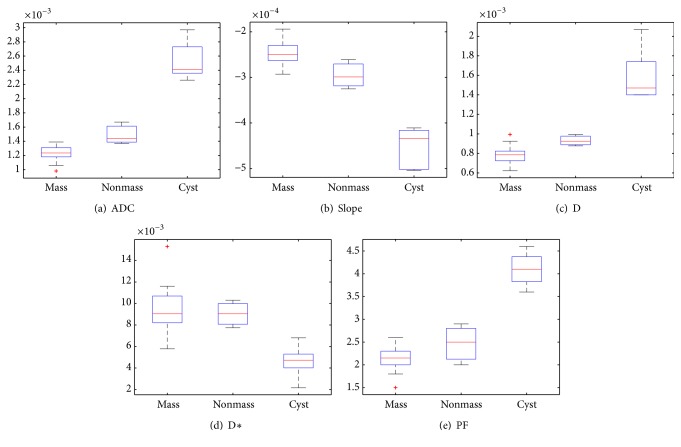
Boxplots of the median values of different types of breast tissue, based on (a) ADC, (b) slope, (c) D, (d) D*∗*, and (e) PF. The horizontal solid red lines within each box represent the median value.

A boxplot is a convenient way of graphically depicting groups of numerical data through their quartiles. Boxplots can show the minimum (Q1), the medium (Q3), interquartile range (interquartile range = Q3 – Q1), and maximum values. The boxplot in Figure 8 illustrates the distribution for all 25 patients of biexponential median values for (a) ADC, (b) slope, (c) D, (d) D*∗*, and (e) PF.

In practice, the histological features of parameter* D* in IVIM are similar to those of parameter* ADC* in DWI. However, it is better to use parameter* D* than to use the parameter* ADC* because it can accurately demonstrate true diffusion without being affected by perfusion-related diffusion. In this study, we also found that parameter* D* has significant differences between malignant tumors and cysts, so the* D* value in IVIM can effectively complement existing traditional DCE-MRI and DWI to distinguish between malignant breast tumors and cysts. The mean* D* value of cysts is higher than the mean* D* value of malignant tumors, close to the result of Ma et al. [31]. Inconsistent parameter results may be due to differences in the number and distribution of low and high* b* values used in IVIM. From the experimental results, we also found that the* D∗* and f values of benign and malignant breast lesions have relatively low effects, so the* D* parameter is given a higher weight in the part of the detection algorithm decision tree. One of the main limitations of our study is that the number of patients is not large enough, including variety of diseases. Second, the selection and appropriate number of* b* values for breast IVIM are still unknown.

Currently DCE-MRI is still one of the most important and mature methods in breast lesion imaging. Although DCE-MRI has high sensitivity for detecting breast cancer, its disadvantage is that it requires injection of contrast agents. However, the significant association between gadolinium-based contrast agents and the incidence of nephrogenic systemic fibrosis (NSF) in patients with advanced renal disease has been reported. DWI is a noninvasive method that uses magnetic resonance imaging to observe the diffusion of water molecules in living tissue. Current results show that IVIM-DWI helps to understand tissue characteristics and distinguish between benign and malignant lesions. Therefore, DWI may have a role as an alternative diagnostic technique for detecting breast lesion without the need of contrast agent.

## 5. Conclusions

In this paper, hyperspectral imaging technique was developed to detect and classify breast tissue lesions, which can be implemented in two stages. The first stage is the detection of the breast tissue, followed by the classification in the second stage. This study makes several contributions to the breast imaging process. First, the most important contribution is to process IVIM-DW images as multispectral images, which can be further extended to hyperspectral images by BEP. Second, an unsupervised hyperspectral target detection algorithm, ATGP, was applied to the expanded IVIM-DW images to determine potential lesion pixels, which can be used as the desired target knowledge for the follow-up target detection algorithm, KCEM, to locate suspected areas of breast cancer lesion tissues. Third, a thresholding technique was used to extract the lesion areas. Fourth, the detected lesion pixels were used to calculate five parameters: ADC, D, D*∗*, PF, and slope via the biexponential model specified by (4). The calculated values of ADC, D, D*∗*, PF, and slope were ultimately input into a decision tree using histogram analysis to classify the detected breast lesion tissues into three categories: malignant tumor (central and peripheral tumor), cysts, and normal tissue. It is our belief that the work presented in this paper is the first to process breast IVIM-DW images as hyperspectral images. Our findings show that hyperspectral imaging techniques could be used to detect breast lesion tissues. We also believe that this work is the first to take advantage of a biexponential model to classify breast lesion tissue by using the five parameters of ADC, D, D*∗*, PF, and slope. This study was limited by its small sample size and it did not employ multiple comparison correlations. Therefore, the usefulness of the IVIM parameters for lesion differentiation requires further investigation.

## Figures and Tables

**Table 1:** Monoexponential analysis (i.e., ADC), IVIM parameters (i.e., D, D*∗*, and PF), and slope in one case of malignant breast cancer cases.

Slice	ADC (10^−3^ mm^2^/s)	slope	D (10^−3^ mm^2^/s)	D^*∗*^ (10^−3^ mm^2^/s)	PF (%)
10	1.34	-0.29	0.743	9.21	26
11	1.24	-0.271	0.737	8.8	23
12	1.21	-0.258	0.732	8.57	22
13	1.2	-0.245	0.718	9.88	22
14	1.25	-0.243	0.741	10.18	23
15	1.29	-0.251	0.771	10.15	23
16	1.31	-0.253	0.778	10.09	24
17	1.26	-0.248	0.761	10.15	22
18	1.18	-0.242	0.734	10.15	20
19	1.15	-0.239	0.707	10.15	20
20	1.15	-0.239	0.68	10.13	21
21	1.22	-0.248	0.696	10.31	23
22	1.21	-0.252	0.702	10.05	23

Average	1.23	-0.252	0.731	10.08	22

**Table 2:** The average values of the monoexponential model (i.e., ADC), IVIM parameters (i.e., D, D*∗*, and PF), and slope analysis of different breast tissues in our experimental case.

	ADC (10^−3^ mm^2^/s)	slope	D (10^−3^ mm^2^/s)	D^*∗*^ (10^−3^ mm^2^/s)	PF (%)
Central Area of the Tumor	1.23 (±0.9)	-0.24 (±0.2)	0.76 (±0.6)	9.38 (±1.1)	21.5 (±1)
Peripheral Area of the Tumor	1.5 (±0.13)	-0.3 (±0.3)	0.93 (±0.5)	9.02 (±1.03)	25.3 (±3)
Cyst	2.54 (±0.25)	-0.45 (±0.4)	1.59 (±0.08)	4.62 (±0.09)	41.1 (±1)
Normal Tissue	1.86 (±0.25)	-0.37 (±0.3)	1.14 (±0.21)	5.98 (±0.21)	31.1 (±2.8)

## Data Availability

The data used to support the findings of this study are included within the article.
